# Synaptic Role in Facilitating Synchronous Theta Oscillations in a Hybrid Hippocampal Neuronal Network

**DOI:** 10.3389/fncom.2022.791189

**Published:** 2022-02-04

**Authors:** Zilu Liu, Qingyun Wang, Fang Han

**Affiliations:** ^1^Department of Dynamics and Control, Beihang University, Beijing, China; ^2^College of Information Science and Technology, Donghua University, Shanghai, China

**Keywords:** E-I network, network dynamics, synchronization, synaptic coupling, theta oscillations

## Abstract

Theta rhythms (4–12 Hz) in the hippocampus are thought to be associated with cognitive functions such as memory processing and spatial navigation. Rhythmic oscillations in the neural system can be induced by synchronization of neural populations, while physiological mechanisms for the emergence, modulation, and regulation of such rhythms are not fully understood. Conceptual reduced models are promising in promoting current understandings toward neural synchronization because of high computational efficiency, while they appear less straightforward in biological relevance. In this study, we use a hybrid E-I network as a conceptual model of the hippocampus to investigate the dynamics of synchronous theta oscillations. Specifically, experimentally constrained Izhikevich neurons and preferential connections among neural groups specific to hippocampal CA1 are incorporated to enhance the biological relevance of the model network. Based on such a model, synaptic factors related to the balance of network excitation and inhibition are the main focus of present study. By careful parameter exploration, the distinct role of synaptic connections in theta rhythm generation, facilitation of synchronization, and induction of burst activities are clarified. It is revealed that theta rhythms can be present with AMPA mediated weak E-I couplings, or with strong NMDA current. Moreover, counter-inhibition, namely inhibition of inhibition, is found effective in modulating the degree of network synchronization, while has little effect on regulating network frequency in both regimes. Under pathological considerations where the effect of pyramidal sprouting is simulated, synchronized burst patterns are observed to be induced by elevated recurrent excitation among pyramidal cells. In the final part, we additionally perform a test on the robustness of our results under heterogeneous parameters. Our simulation results may provide insights into understanding how brain rhythms are generated and modulated, and the proposed model may serve as a useful template in probing mechanisms of hippocampal-related dynamics.

## 1. Introduction

Rhythmic oscillations of neural ensembles play an important role in information processing in the brain (Buzsaki, 2004). Studying the underlying mechanisms of brain rhythms is considered fundamental to understanding higher brain functions. The hippocampus, as one of the most extensively studied mammalian brain regions, is hypothesized to be the structural substrate for many functional-related brain rhythms. Rhythmic oscillations in the hippocampus can be physiological or pathological, such as theta rhythms and epileptic seizures (Isomura et al., 2008). Hippocampal theta oscillations (4–12 Hz) have long been observed in animal experiments and are assumed to be functionally linked to memory processing during REM sleep and spatial navigation during exploratory behaviors (Huxter et al., 2003). Such associations were also reported in human studies (Lega et al., 2012). Extensive efforts have tried to link the role of theta rhythm with higher brain function through empirical data (Hasselmo, 2005; Korotkova et al., 2018). Therefore, analyzing these rhythms can provide in-depth knowledge about how the hippocampal-related systems work under normal and pathological conditions, of which the mechanisms are still not fully understood (Buzsáki, 2006).

Spiking neuronal networks can provide insights into how oscillatory activities can occur through network emergent behavior such as synchronization (Arenas et al., 2008). On a mesoscopic level, rhythms occur through the synchronization of groups of spiking neurons, as a result of interactions between intrinsic neuronal properties and network connectivity under both normal and pathological states. These neuronal networks usually consist of excitatory and inhibitory neurons coupled by chemical or electrical synapses, serving as a substructure for network oscillations to occur. How rhythmic activities emerge in a coupled E-I network has been a topic for a long time. Conceptually, a principle for the generation of gamma rhythm in E-I networks has been established, which is known as the Pyramidal Interneuron Network Gamma (PING) mechanism (Kopell et al., 2010). Several internal or external factors were further linked to the modulation and regulation of PING network dynamics (Rich et al., 2017; Gu et al., 2021). As for network connectivity, many works simply treat the network topology in some mathematical formats such as random graphs (Qin et al., 2018). While this simplification is mainly necessitated by the lack of data (De Schutter, 2008), it can simplify the analysis to allow more hypotheses to be tested and may lead to some general governing rules (Zhou et al., 2006).

Specific to hippocampal theta rhythm, a conductance-based network consisting of multi-compartmental pyramidal and GABAergic interneurons was capable of generating robust theta rhythm as a result of interactions between PY and IN (Kiss et al., 2006). Even at a higher level of biological realism, a full-scale data-driven CA1 network aggregated by Hodgkin-Huxley type of neurons was constructed and provided implications of the role of inhibitory diversity in rhythm generation (Bezaire et al., 2016). While biophysically-detailed models like HH based networks are frequently employed to interpret neurophysiological phenomena, the overlarge parameter space and huge demand in computing resources will make model exploration difficult, thus limiting the application value of these models (Traub et al., 1993; Morgan and Soltesz, 2008). As alternatives to these detailed models, phenomenological oscillators can be used to mitigate the computation burden of network simulation (Izhikevich, 2004). By summarizing the complex ionic exchanges into one controlling parameter and introducing a reset mechanism, the Izhikevich neuron preserves the most important aspect of the upstroke dynamics in HH neurons and can replicate many realistic computational properties through proper parameter tuning while free of heavy computation burden (Izhikevich, 2007). One limitation of these models is lack of biological relevance. But this can partly be compensated by additional biological constraints, as suggested in Lytton et al. (2008). Previous works have been devoted to quantifying the diverse phenotypes of hippocampal neurons by fitting Izhikevich neurons with electrophysiological data (Ferguson et al., 2013; Venkadesh et al., 2019). Investigations of rhythmic behavior on such type of network were performed in an experimentally constrained Izhikevich network composed of hippocampal pyramidal and parvalbumin-positive neurons (Ferguson et al., 2017), and specific intrinsic and synaptic factors were identified to be most contributing to theta rhythm generation.

Motivated by previous works of biological-relevant phenomenological networks, this study aims to investigate the rhythmic dynamics exhibited in a conceptual hippocampal CA1 network in the theta range with hybrid cells and connectivity. Specifically, four populations of experimentally constrained Izhikevich neurons acknowledged to be relevant in hippocampal rhythmic activities are considered to enhance the biological relevance of the model. The hybrid excitatory and inhibitory network includes non-bursting and bursting Pyramidal cells (PY), fast-spiking parvalbumin-positive basket cells (PV+), and low-threshold spiking somatostatin-positive cells (SOM+). Network topology is considered random with preferential connectivity existing between PV+ and PY subpopulations. This connectivity is inspired by recent neurobiological evidence that a preferential connectivity pattern between PY and PV+ cell population is present, with pyramidal neurons from the superficial and deep layers of hippocampus radial axis making non-uniform connections to PV+ cells (Lee et al., 2014).

The network dynamics of interest in this study involves theta-band synchronized spike and burst activities. Synaptic couplings between different neural populations are explored to clarify their contribution to these rhythmic activities. Parameter regimes that support the emergence of theta oscillations are identified through careful parameter exploration. The explorations are performed under physio- and pathological considerations, which leads to the observation of synchronized spike and burst dynamics among the hybrid pyramidal neurons, respectively. We use quantitative measures to characterize the degree of synchronization and burst activities. For each type of network dynamics, we focus on the crucial synaptic factors responsible for network transition dynamics by regulating or modulating theta-band synchronization. Finally, the robustness of our results in respect to parameter heterogeneity is reconfirmed by replacing the identical parameters with distributed values.

## 2. Methods

Izhikevich neurons of four cell types are used to form a hybrid E-I network, including PY_NB_, PY_BT_, PV+, and SOM+. Though the network is constructed in a conceptual way, some neurophysiological details regarding neuronal intrinsic properties and network connectivity are considered to enhance its biological relevance to realistic systems. As for couplings, chemical synapses are explicitly modeled for neural communication and are used to make random connections between neural populations. The network consists of 800 excitatory and 200 inhibitory neurons with each excitatory (inhibitory) subpopulation being equal in numbers. More details on network structure are described as follows.

### 2.1. Cell-Type Specific Izhikevich Neuronal Model

The modified form of the Izhikevich neuronal model is employed as its parameters can be tuned to approach neuron's realistic behavior. The two-dimensional phenomenological model is given by the following equations (Izhikevich, 2007):

(1) $$\begin{array}{r} \begin{array}{c} C_{\text{m}}\frac{\text{d}v}{\text{d}t}=k\left(v-v_{\text{r}}\right)\left(v-v_{\text{t}}\right)-u+I_{\text{syn}}+I_{\text{tonic}}\\ \;\frac{\text{d}u}{\text{d}t}=a\left(b\left(v-v_{\text{t}}\right)-u\right)\\ \;if\;v=v_{\text{peak}},\;then\;v=v_{\text{min}},\;u=u+d \end{array} \end{array}$$

where state variable *v* denotes the membrane potential and *u* is the slow recovery process representing a summed effect of ionic inward and outward currents across the membrane. Dynamics shaping parameters *k*, *a*, *b*, and *d* together with electrophysiology-related *C*_m_, *v*_t_, *v*_r_, *v*_min_, and *v*_peak_ are adapted from various published resources for each cell type. *I*_tonic_ is a constant current identically applied to all PY cells, mimicking the incoming excitatory drive from external sources and fixed at 70 pA to ensure sufficient network driving force, while interneurons are not driven by any external input. *I*_syn_ represents the sum of synaptic currents from other neurons in the network, and is discussed in the next section.

**Figure 1:**
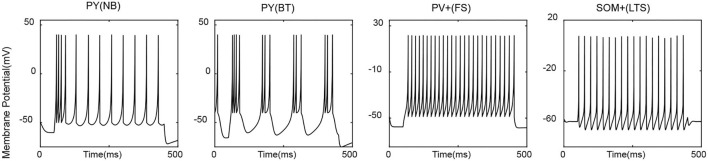
Exemplary firing pattern of each cell type in response to a tonic input = 225 pA. NB, non-bursting; BT, bursting; FS, fast-spiking; LTS, low-threshold spiking.

Parameters selected for each type of neuron aim to make it display type-specific firing patterns (Figure 1). PY cells are typically non-bursting ones, but bursting types do exist, and there is evidence that PYs from the deep layer along the CA1 radial axis are more prone to show burst dynamics than superficial PYs do (Cembrowski and Spruston, 2019). Therefore, both non-bursting and bursting PYs are included to be indicative of various dynamics among CA1 pyramidal cells. The non-bursting PY neuron is taken from Izhikevich's book (Izhikevich, 2007), based on which the bursting PY is obtained with a slight modification. For inhibitory neurons, peri-somatic PV+ cells are typically of the GABAergic fast-spiking type and are the main contributors of fast inhibition to corresponding principal cells. Dendritic inhibitory SOM+ cells are mainly of the low-threshold spiking type that is able to generate post-inhibitory rebound spike in response to a hyperpolarized input (Liguz-Lecznar et al., 2016). Parameters for PV+ and SOM+ were picked from hippocampome.org (Venkadesh et al., 2019), a database providing data-fitted Izhikevich models for various phenotypes of hippocampal neurons. We modified them a bit without changing the major dynamics to make the range of firing rate agreeable to the frequency-input curve in published literature (Ferguson, 2015).

### 2.2. Network Connectivity With Preferential Connections

**Figure 2:**
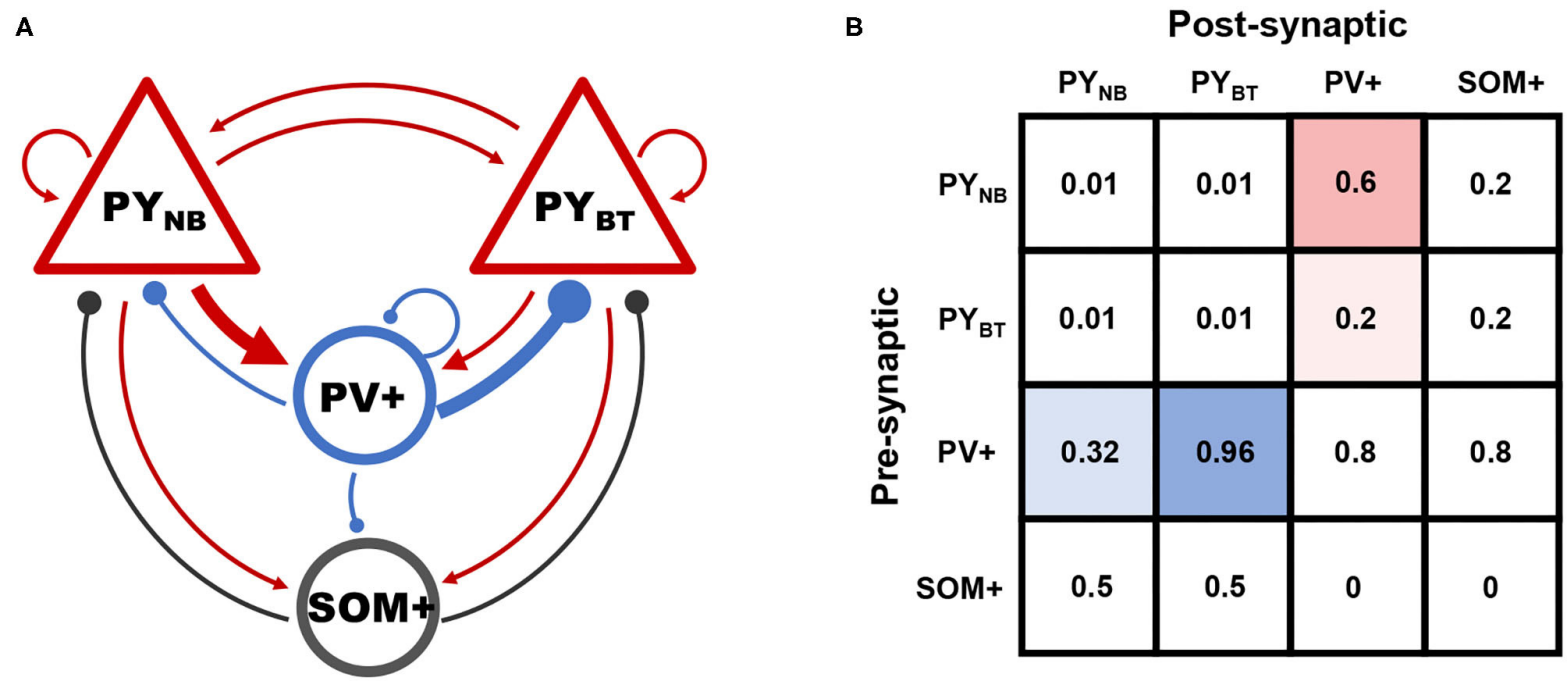
**(A)** Schematic of connectivity among different groups. Arrows and solid circles indicate excitatory and inhibitory effects, respectively. **(B)** The matrix of connection probability. Preferential connections between subpopulations of PY and PV+ are highlighted by blue and red shadows.

Network connectivity is assumed to be random, while preferential connections exist between subpopulations of PY and PV+ cells. Evidence is that fast-spiking PV+ cells provide three times more synaptic current to deep-layer PYs than superficial ones, while superficial PYs contrarily connect three times more often to PV+ cells (Soltesz and Losonczy, 2018), so that a biased loop between PY and PV+ subpopulations is formed. We attribute this preferential connectivity to distinct connection probabilities between the two groups (Figure 2).

Connections between other neural populations are determined based on biological facts that: (a) peri-somatic PV+ cells connect rather densely to principal neurons, (b) recurrent connections among PYs are sparse in CA1, (c) interactions between SOM+ and PY are weaker than that in PV-PY circuit as SOM+ cells mainly make contacts at the dendrite, and (d) counter-inhibition provided by PV+ cells was reported to be significant in the hippocampus (Paz and Huguenard, 2015).

Synaptic interactions between neurons are described by the Gradual Rise model (Börgers, 2017a):

(2) $$\begin{array}{r} \begin{array}{c} I_{\text{syn}}=g_{\text{syn}}s_{\text{pre}}\left(t\right)\left(v_{\text{rev}}-v_{\text{post}}\right)\\ \frac{\text{d}s}{\text{d}t}=q\frac{1-s}{\tau_{\text{r}}}-\frac{s}{\tau_{\text{d}}}\\ \frac{\text{d}q}{\text{d}t}=\frac{1+tanh\left(v/10\right)}{2}\frac{1-q}{\tau_{\text{r,q}}}-\frac{q}{\tau_{\text{d,q}}} \end{array} \end{array}$$

where *s* and *q* are synaptic gating variables. Just similar as in real biological systems, three types of chemical receptors including AMPA, NMDA, and GABAa are considered. AMPA and GABAa mediated activities typically respond fast to incoming signals with small time constants, while that of NMDA usually act on a much slower time scale. Specifically, for NMDA receptors, *I*_syn_ is modeled in a slightly different formulation as suggested by Izhikevich (Izhikevich, 2004):

(3) $$\begin{array}{r} I_{\text{NMDA}}=g_{\text{NMDA}}\frac{{\left[\left(v+80\right)/60\right]}^{2}}{1+{\left[\left(v+80\right)/60\right]}^{2}}s_{\text{pre}}\left(t\right)\left(v_{\text{rev}}-v_{\text{post}}\right) \end{array}$$

The reversal potential *v*_rev_ is assigned to be 0 mV for excitatory AMPA and NMDA, and −70 mV for inhibitory GABAa.

Synaptic conductance *g* is the main parameter to be explored in the following simulations. We consider *g* varying according to the receptor type between pre- and post-synaptic populations. For example, *g*_AMPA_ represents the synaptic coupling strength from excitatory to inhibitory population mediated by AMPA receptors, and is assumed to be identical for all connections of the same type in most cases except for the last section of results, where we perform additional tests on the influence of parameter heterogeneity.

Values for the aforementioned parameters are summarized in Tables 1, 2.

**Table 1:** Neuronal parameters.

	**k**	**a**	**b**	**d**	***C***_**m**_	***v***_**r**_	***v***_**t**_	***v***_**min**_	***v***_**peak**_	***I***_**tonic**_	**Source**
PY_NB_	0.5	0.01	5	50	50	−60	−45	−50	40	70	Izhikevich, 2007
PY_BT_	0.5	0.01	5	60	60	−60	−45	−40	40	70	modified
PV+	1.19	0.005	0.22	2	75	−57.63	−35.53	−48.7	21.72	0	Venkadesh et al., 2019
SOM+	4.47	0.069	74.3	299	73	−60	−56.41	−58.16	7.99	0	Venkadesh et al., 2019

**Table 2:** Synaptic parameters.

	**τ_r_**	**τ_d_**	**τ_r,q_**	**τ_d,q_**	***v***_**rev**_	***g***_**ee**_	***g***_**ei**_	***g***_**ie**_	***g***_**ii**_
AMPA	0.5	3	0.1	0.17	0	**var**	**var**	-	-
NMDA	5	150	0.1	1.01	0	0.1	**var**	-	-
GABAa	0.5	3	0.1	0.17	−70	-	-	**var**	**var**

### 2.3. Measures

Network output is computed as the average membrane potential of all pyramidal neurons (both PY_NB_ and PY_BT_) to approximate Local Field Potential (LFP). The simulated signal is evaluated in terms of spectral and temporal aspects to inform its oscillatory property. Since we mainly focus on global activity located in the theta range, network dominant frequency is computed first by finding the spectral peak of the fast Fourier transform of the simulated LFP. The overall synchronization of pyramidal neurons is manifested by the degree of field potential fluctuations, since a larger deflection of LFP will only be achieved when more neurons fire almost simultaneously. To quantify this fluctuation of network output, we adopt the statistical metric standard deviation (SD) to reflect the oscillation in field potential. The simulated LFP first needs to be normalized by subtracting its time average to guarantee comparable metrics. SD is defined as informed in probabilistic theory:

(4) $$\begin{array}{r} \text{SD}=\sqrt{\frac{\sum_{i=1}^{n}{\left(x_{\text{i}}-\bar{x}\right)}^{2}}{n}} \end{array}$$

where *n* denotes the total number of data points in the time series. *x*_*i*_ and $\bar{x}$ refers to each sample point and average of data, respectively. In this way, a higher level of network oscillation corresponding to large-amplitude global activities will result in a larger SD. When neurons fire almost randomly, in which sense no rhythmic activity is formed so that the corresponding SD will be small. In this study, we think an output with SD <2.5 exhibits no rhythmic activity. Under an SD of such level, the network does not fire synchronously all the time during the simulation, while the largest SD in all the simulations is around 12. We have to say that since network dynamics varies rather smoothly, the threshold is only used to make a vague distinction between rhythmic and non-rhythmic activities. Therefore, result interpretations have to be made a bit far from these non-existing boundaries.

Under some parameter sets, the network will exhibit synchronized burst dynamics. We adopt the coefficient of variation (CV) to further differentiate the burst dynamics from spike patterns under synchronization scenarios. The coefficient of variation of a single neuron is given by:

(5) $$\begin{array}{r} {\text{CV}}_{\text{i}}=\frac{\sigma_{{\text{ISI}}_{\text{i}}}}{{\bar{\text{ISI}}}_{\text{i}}} \end{array}$$

ISI_*i*_ denotes the inter-spike-interval sequence of a specific neuron i, and σ_ISI_i__ is its standard deviation. The value of CV is obtained by average over all the pyramidal neurons.

(6) $$\begin{array}{r} \text{CV}=\frac{1}{N}\overset{N}{\underset{i=1}{\sum }}{\text{CV}}_{\text{i}} \end{array}$$

When CV surpasses 0.5, we think the network produces a bursting pattern as suggested in Börgers (2017b).

### 2.4. Simulation Settings

Each simulation was run for 5 s in total, and the last 3 s of activity were saved to eliminate transients. Variable *v* was randomly initialized around the resting membrane potential of each cell type, and other variables were all started from zero. Numeric simulations were implemented using the second-order mid-point method with a time step of 0.04 ms. All the simulating and analyzing processes were done in MATLAB.

## 3. Results

Synaptic couplings are studied for their roles in promoting synchronous theta oscillations. The explorations are arranged under physio- and pathological settings respectively. Both synchronized spike and burst dynamics are observed in the present study. We first coarsely identify the parameter regime that supports network global activities to be located in the theta band. Then, the distinct roles of couplings in regulating and modulating network rhythms are investigated, by which means we identify how varying parameters will lead to transitions of network dynamics. In the final part, we perform additional tests on the robustness of our results when parameter heterogeneity is considered.

### 3.1. Explorations Under Physiological Settings: Synchronized Spike Patterns

CA1 pyramidal neurons are reported to be sparsely connected with each other (Witter, 2010). Therefore, under physiological settings, we do not consider the effect of recurrent excitation on network dynamics to reduce the number of degrees of freedom of parameter space (by assuming *g*_ee_ = 1). Both computational and experimental evidence has suggested the significance of inhibitory effects on theta rhythm generation (Wulff et al., 2009), which motivates us to focus on the inhibitory-related parameters *g*_ei_, *g*_ie_, and *g*_ii_, denoting the excitation and inhibition between E-I groups, and counter-inhibition provided by PV+ cells, respectively. In the present study, *g*_ei_ refers to either *g*_AMPA_ or *g*_NMDA_ according to the receptor types.

**Figure 3:**
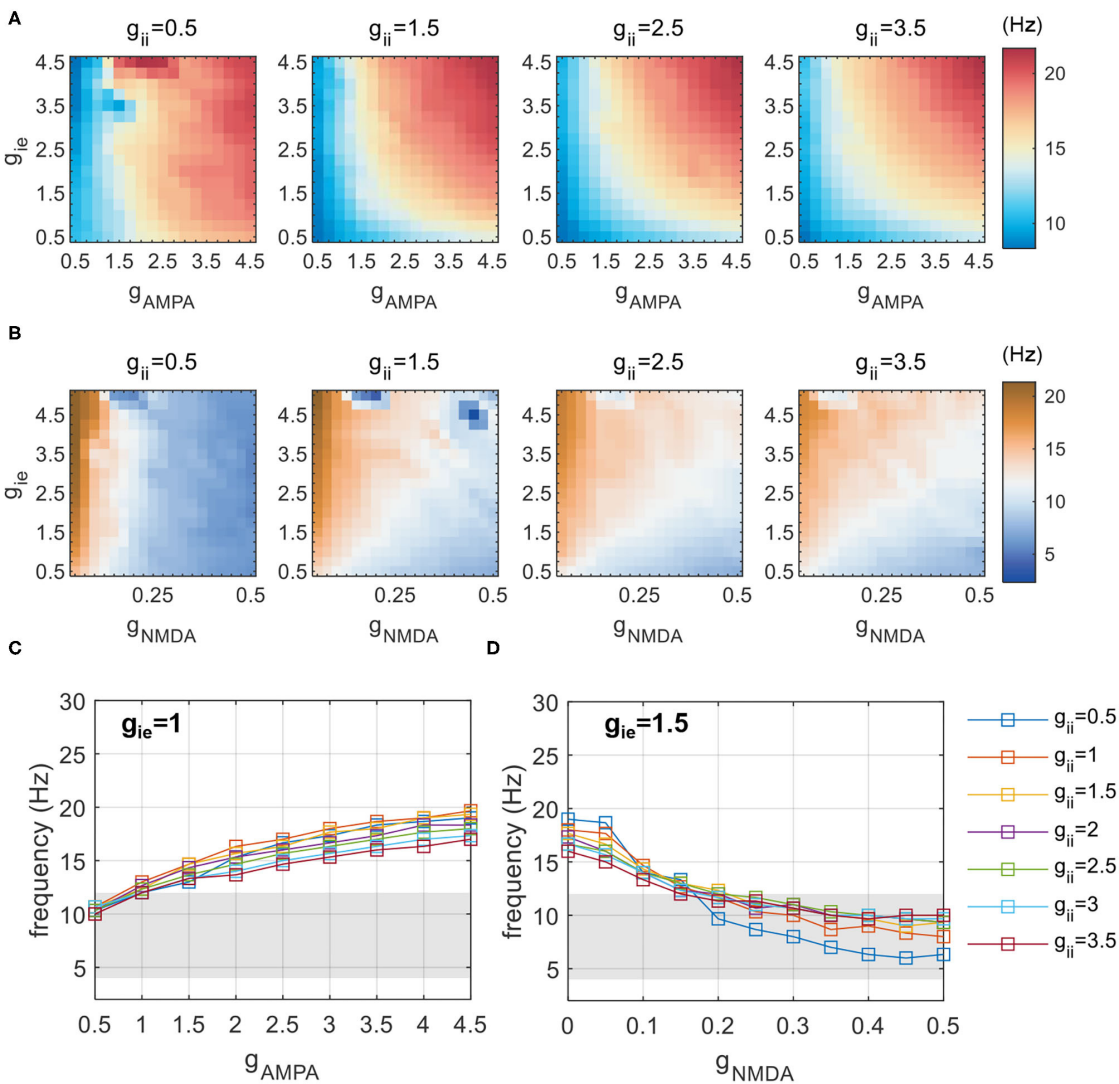
Network frequency influenced by inhibitory-related synaptic parameters (*g*_AMPA_,*g*_NMDA_,*g*_ie_, and *g*_ii_). **(A)** Network frequency obtained by a coarse scanning of [*g*_AMPA_, *g*_ie_, *g*_ii_] to determine the parameter range that constrains activities in the theta range with *g*_NMDA_ = 0.1. **(B)** A similar exploration of [*g*_NMDA_, *g*_ie_, *g*_ii_] with *g*_AMPA_ = 1.5. **(C)** The frequency increases with varying *g*_AMPA_ at distinct *g*_ii_, and theta activities are found to be present with *g*_AMPA_ < 1. **(D)** The frequency decreases with varying *g*_NMDA_ at distinct *g*_ii_, and theta activities are found to be present with *g*_NMDA_ > 0.2. The shaded area denotes the frequency band of theta.

We first identify the conditions supporting the existence of theta rhythms with *g*_NMDA_ = 0.1. This is realized by a coarse exploration of [*g*_AMPA_, *g*_ie_, *g*_ii_] in the range of [0.5,5]. Figure 3A shows the network frequency on the plane of *g*_AMPA_ and *g*_ie_, with *g*_ii_ varying from 0.5 to 3.5 additionally. As *g*_ii_ remains constant in each subplot, network frequency is mainly controlled by the strength of reciprocal couplings between excitatory and inhibitory populations (*g*_AMPA_ and *g*_ie_). It reveals that a simultaneous elevation of reciprocal couplings between E-I groups can produce faster activities, so that slow theta rhythms are supported in the weak coupling regime (*g*_AMPA_, *g*_ie_ < 1). Meanwhile, the frequency pattern is almost invariant with respect to *g*_ii_, as shown by the heatmaps of Figure 3A from left to right. This invariance is more clearly illustrated in Figure 3C. The largely overlapped lines suggest that network frequency is little influenced by the variation of *g*_ii_, while rises monotonically at the increase of fast excitatory-inhibitory couplings. This indicates that counter-inhibition provided by PV+ cells exhibits limited impacts on regulating network activities.

**Figure 4:**
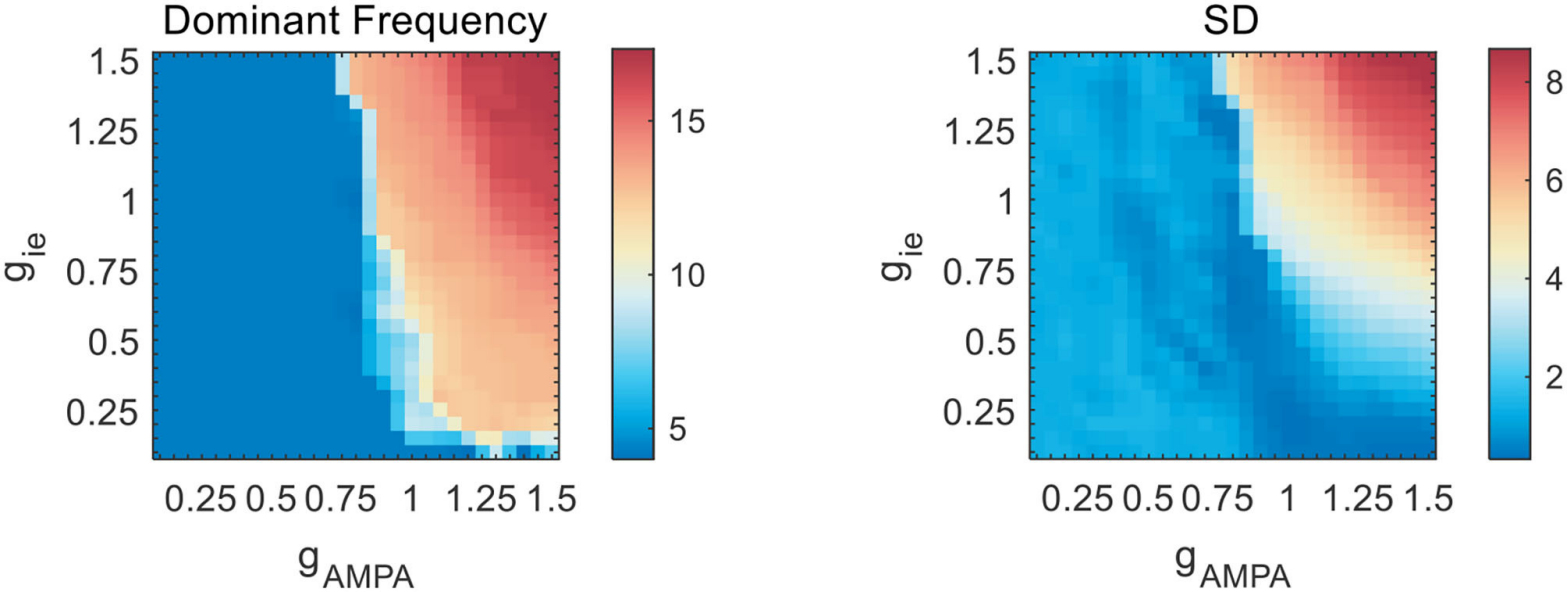
Network frequency and SD on the *g*_AMPA_-*g*_ie_ plane without slow NMDA current. As *g*_AMPA_ increases, the network exhibits a sudden transition into fast oscillations beyond theta range.

As the slow NMDA current assumed in previous simulations is a potential contributor to the observed theta oscillations, we redo the above explorations without NMDA current. The results are shown in Figure 4. It is clear that, as reciprocal E-I couplings become stronger, the network exhibits a sudden transition into rhythmic activities with the network frequency beyond theta range. Such dynamic patterns are also weakly influenced by *g*_ii_ (not shown). These results suggest that the observed theta oscillations in the weak E-I coupling regime is largely slowed down by the presence of NMDA current. To further demonstrate the slowing role of NMDA, we additionally perform an exploration of slow E-I couplings with *g*_AMPA_ = 1.5. The simulation results are presented in Figure 3B. It is found that the slowing down effect of NMDA is obvious but becomes less significant when *g*_ie_ becomes stronger, which probably means an activation of E(AMPA)-I coupling that drives fast network activities. Theta oscillations can be found with large *g*_NMDA_ and relatively small *g*_ie_. Such a frequency pattern is also insensitive to *g*_ii_, as given by Figure 3D.

By coarse scanning of inhibitory-related synaptic parameters, we notice that theta activities can be present in two regimes of couplings: one is with AMPA mediated weak E-I coupling (*g*_AMPA_, *g*_ie_ ∈ [0, 1]) and another is with strong NMDA current (*g*_NMDA_ ∈ [0.2, 0.5], *g*_ie_ ∈ [0.5, 2.5]). Network frequency in both regimes is almost insensitive to the variation of *g*_ii_. Therefore, in the following simulations we will only focus on network dynamics in these two regimes of theta-band activities.

**Figure 5:**
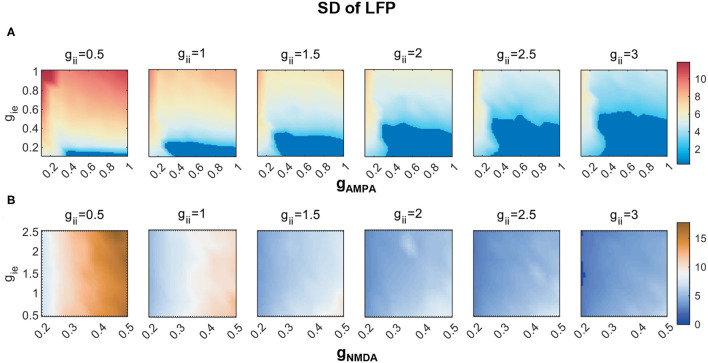
The degree of network synchronization modulated by *g*_ii_) in **(A)** the weak AMPA regime (*g*_AMPA_, *g*_ie_ ∈ [0, 1]) and **(B)** the strong NMDA regime (*g*_NMDA_ ∈ [0.2, 0.5], *g*_ie_ ∈ [0.5, 2.5]). The degree of synchronization is indicated by SDs of LFP. Activities thought to have no rhythms are marked in dark blue, of which the SD is less than 2.5.

Rhythmic oscillations require neurons to fire synchronously. As has been mentioned in the Measures section, we adopt SD to imply the degree of synchronization of network activities. In the weak E-I coupling regime, a systematic measuring of SD under different combinations of [*g*_AMPA_, *g*_ie_, *g*_ii_] is given by Figure 5A. The dark blue region is assigned to represent non-rhythmic activities where SD is less than 2.5, and is present at areas of high *g*_AMPA_ and low *g*_ie_. Within each subplane, we can observe that SD varies more dramatically along the vertical than horizontal axis, which indicates that *g*_ie_ has a stronger control on rhythm generation than *g*_AMPA_ does. It is clear that an increase of inhibition onto PY neurons can effectively facilitate network synchronization.

**Figure 6:**
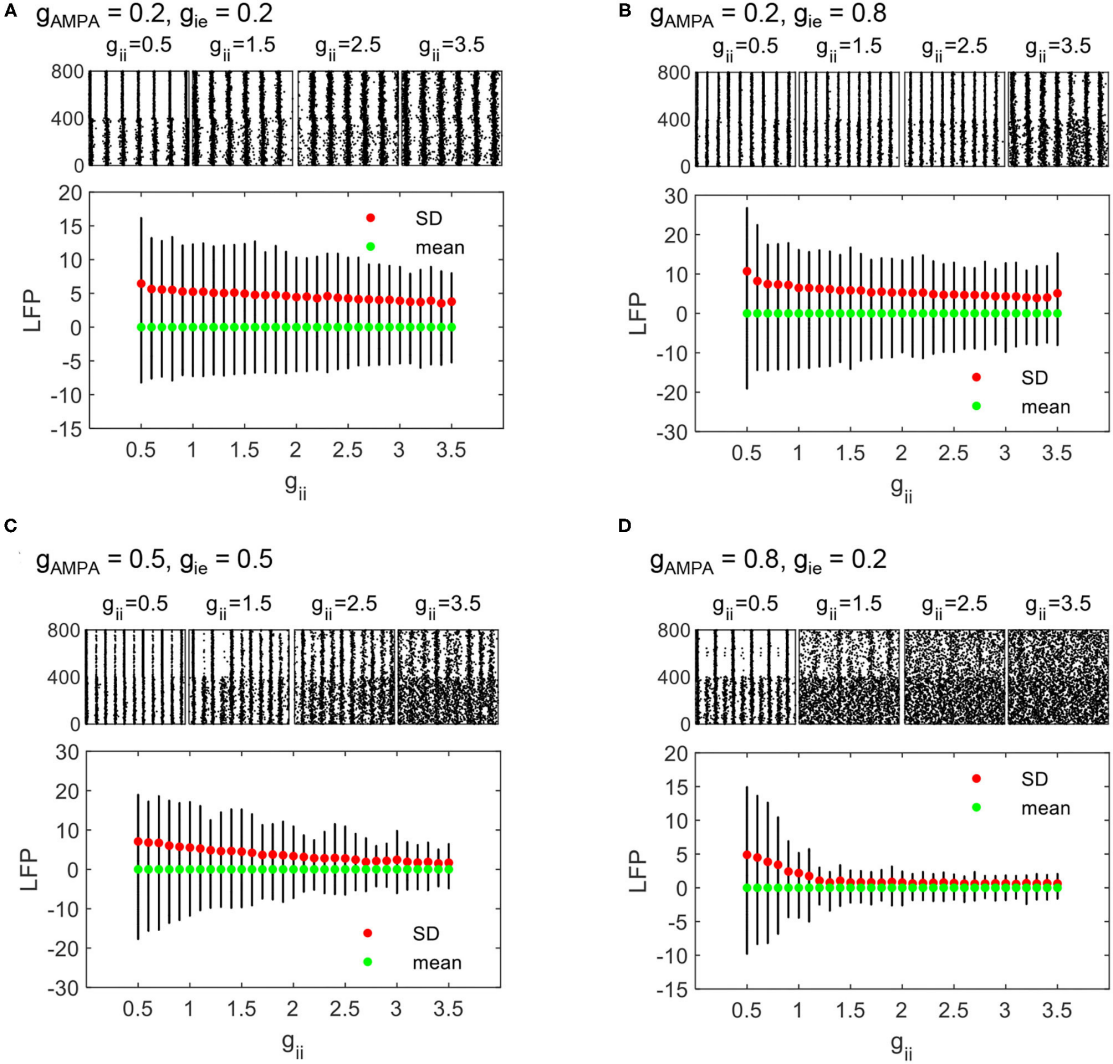
The evolution of LFP fluctuations and sampled firing patterns as *g*_ii_ varies from 0.5 to 3.5. PY_NB_s and PY_BT_ are indexed as 1–400 and 401–800, respectively. The dynamic process is displayed under four different parameter sets: **(A)** *g*_AMPA_ = 0.2, *g*_ie_ = 0.2, **(B)** *g*_AMPA_ = 0.2, *g*_ie_ = 0.8, **(C)** *g*_AMPA_ = 0.5, *g*_ie_ = 0.5, and **(D)** *g*_AMPA_ = 0.8, *g*_ie_ = 0.2.

As *g*_ii_ increases, we can see that the regime of non-rhythmic activities (SD <2.5) expands. Moreover, the overall degree of synchronization is lower. These results indicate that the decline of counter-inhibition can largely facilitate network synchronization. To visualize the synchronizing effect of *g*_ii_ more clearly, raster and LFP fluctuations of four networks with different combinations of *g*_AMPA_ and *g*_ie_ are displayed in respect to varying *g*_ii_, which is given by Figures 6A–D. In each plot, the fluctuation of LFP is mitigated by the increase of *g*_ii_ with decreasing SD (denoted by red dots). Note that the output LFP is normalized to zero to ensure comparable SDs (denoted by green dots). In fact, a decrease in SD is caused by the weakening of network synchronization which is manifested more clearly in the spike raster above, where the synchronized firing pattern of PYs gradually becomes more irregular. Under different parameter sets, the degree of network synchronization to which modulated by *g*_ii_ is different, but the role of *g*_ii_ in modulating network dynamics is generally consistent.

Under the regime of strong NMDA, we notice that network synchronization is quite significant across all parameter combinations, while a similar role of *g*_ii_ in modulating the degree of synchronization is also found (Figure 5B).

### 3.2. Explorations Under Pathological Settings: Synchronized Burst Patterns

Pathological states such as epileptic seizures are thought to be related to an unbalance of network excitation and inhibition. CA1 pyramidal sprouting is one of the pathological changes that can be observed in epileptic tissues, which may serve as a source of network hyperexcitation (Lehmann et al., 2000). In this section, we will examine the effect of pyramidal recurrent excitation (*g*_ee_) on network dynamics. In view of previous results, we have noticed that E-I couplings in the weak regime tend to influence network dynamics jointly rather than independently. Therefore, for clarification purposes, in the following sections, we will perform our simulations over the four particular E-I couplings as in Figure 6.

**Figure 7:**
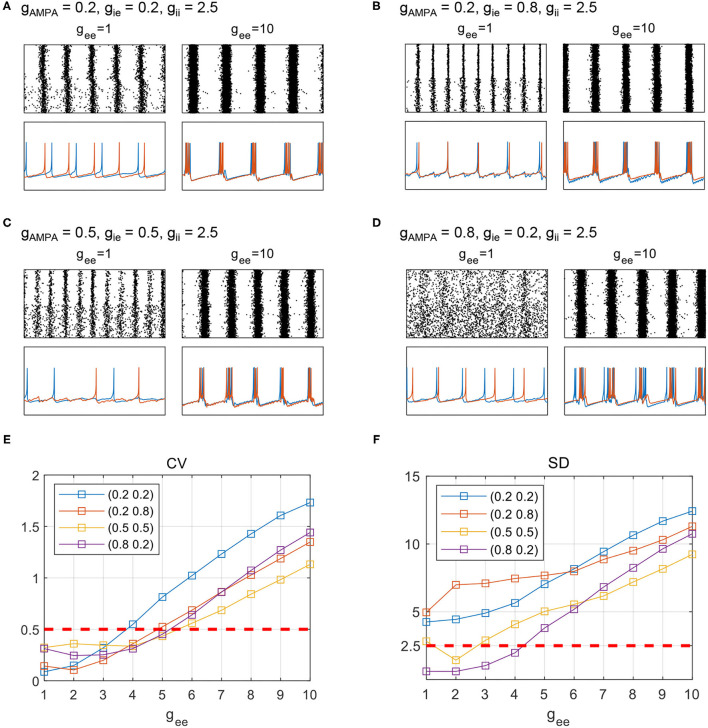
Transition of network states with an enhancement of PY recurrent excitation. **(A–D)** Raster and sampled voltage traces from either of the PY groups. Red and blue traces are sampled from one of the PY_BT_ and PY_NB_ neurons, respectively. The fixed parameter set for each subplot is **(A)** *g*_AMPA_ = 0.2, *g*_ie_ = 0.2, **(B)** *g*_AMPA_ = 0.2, *g*_ie_ = 0.8, **(C)** *g*_AMPA_ = 0.5, *g*_ie_ = 0.5, and **(D)** *g*_AMPA_ = 0.8, *g*_ie_ = 0.2. **(E)**, **(F)**: The evolution of CV and SD under the four parameter sets as *g*_ee_ varies form 1 to 10.

We first investigate the transition of network dynamics under various levels of *g*_ee_, with other synaptic parameters remaining constant. Apart from network synchronized spikes, burst activities are observed when *g*_*ee*_ increases steply, as shown by the network states in Figures 7A–D. Network firing patterns and sampled neuronal voltage traces from PY_NB_ and PY_BT_ groups are displayed together to inform the transition into network burst dynamics. Regardless of the initial states of networks, an enhancement of recurrent excitation can always lead the networks to burst synchronously, which is realized by the burst synchronization of individual neurons. To quantitatively capture the evolution of network dynamics, we further use coefficient of variation (CV) to measure the degree of burst activities. As suggested in Börgers (2017b), we think a particular network exhibits burst activities when CV is over 0.5. The evolution of CV as *g*_ee_ varying from 1 to 10 is depicted in Figure 7E. Consistent with the trend discussed above, the level of CV as an indicator of bursting degree rises and finally surpasses 0.5 somewhere as *g*_ee_ grows gradually. The network with the weakest E-I couplings (*g*_AMPA_ = 0.2, *g*_ie_ = 0.2) experiences the most drastic change in CV in response to the variation of *g*_ee_, and can finally achieve the highest level of burst synchronization. This is clearly reflected in Figure 7A. Though a high level of burst activities always occurs in a synchronized form in the network, the measure of CV is essentially not an indicator for synchronization. Therefore, the evolution of SD is computed as well to additionally illustrate the degree of network synchronization. As is expected, a monotonic increase in SD is observed as *g*_ee_ increases under all parameter sets, which clarifies the role of *g*_ee_ in promoting burst synchronization. Similar spike to burst transition can also be observed in the strong NMDA regime (not shown), while network dynamics is much less sensitive to *g*_ee_ (synchronized burst patterns occur with *g*_ee_ over 50) probably due to the already high level of excitation in network E-I balance.

**Figure 8:**
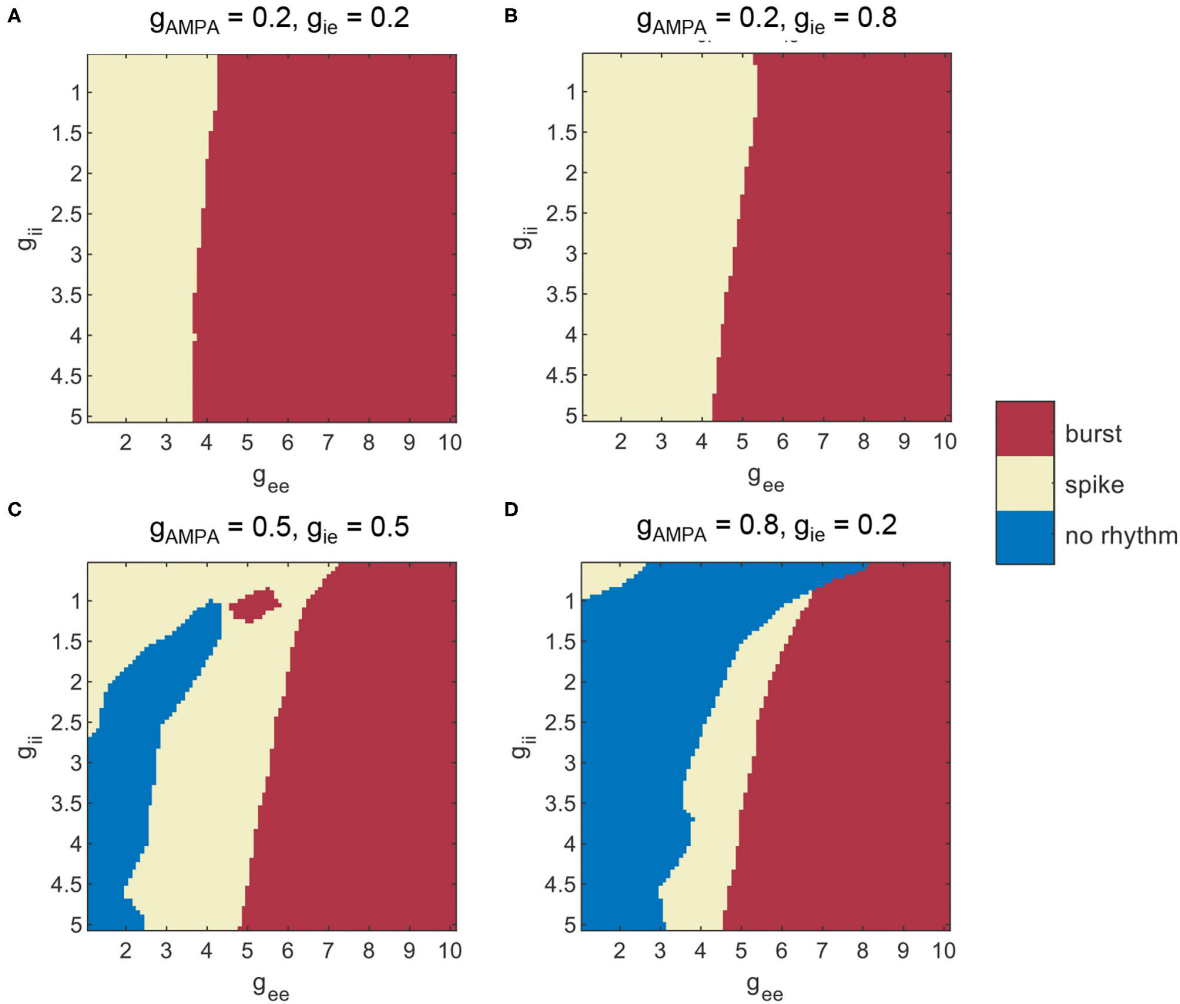
Different patterns of network dynamics mediated by recurrent excitation and counter-inhibition under three levels of E-I couplings. **(A)** *g*_AMPA_ = *g*_ie_ = 0.2, **(B)** *g*_AMPA_ = 0.2, *g*_ie_ = 0.8, **(C)** *g*_AMPA_ = *g*_ie_ = 0.5, **(D)** *g*_AMPA_ = 0.8, *g*_ie_ = 0.2.

As suggested from Section 3.1, counter-inhibition is effective in modulating network synchronization. Therefore, network dynamics is further examined under various combinations of both recurrent excitation and counter-inhibition. The exploration of network dynamics is still performed under the four conditions of E-I couplings, while distinct network states are assigned according to computed SDs and CVs. The corresponding network behavior is classified as burst, spike, and no rhythm activities based on combinations of the two metrics. We first use SDs to differentiate scenarios with or without synchronization. As is mentioned before, synchronous oscillatory activities are thought to be those with SD>2.5, otherwise, they would be considered as non-rhythmic activities. Among the synchronous scenarios, bursting patterns are further differentiated from spike patterns when CV surpasses 0.5. The results obtained under the four particular parameter sets are given by Figures 8A–D, where each type of dynamics is marked in a specific color. Several results are reconfirmed in Figure 8. For example, areas thought to have no rhythmic activities are present in conditions without enough inhibition onto excitatory populations (Figures 8C,D). We can also see that the conclusion drawn from Figure 7 is still applicable to Figure 8, that a larger *g*_ee_ will favor the network to exhibit synchronized burst activities. In addition, the transition into burst dynamics may get easier under a higher level of counter-inhibition since the boundary between spike and burst activities tilts toward a smaller *g*_ee_ as *g*_ii_ increases. However, the role of *g*_ii_ in modulating network dynamics is not that obvious as in Section 3.1. Facilitation of synchronization mediated by the decline of *g*_ii_ can only be observed when *g*_ee_ is weak enough. This is probably due to the strong domination of *g*_ee_ in network E-I balance.

### 3.3. Robustness of Results in Respect to Parameter Heterogeneity

The above results are obtained with the parameter *g* being identical to all the connections of the same type. While in a more biologically realistic setting, parameters are not identical but distributed in a physiological range. It is not sure if the results still remain unchanged when parameter heterogeneity is introduced.

To test the robustness of results in terms of parameter heterogeneity, a uniform distribution of [(1 − σ)*g*, (1 + σ)*g*] is used to generate the random coupling strength for each connection, where σ is the percentage of deviation from the original *g*.

**Figure 9:**
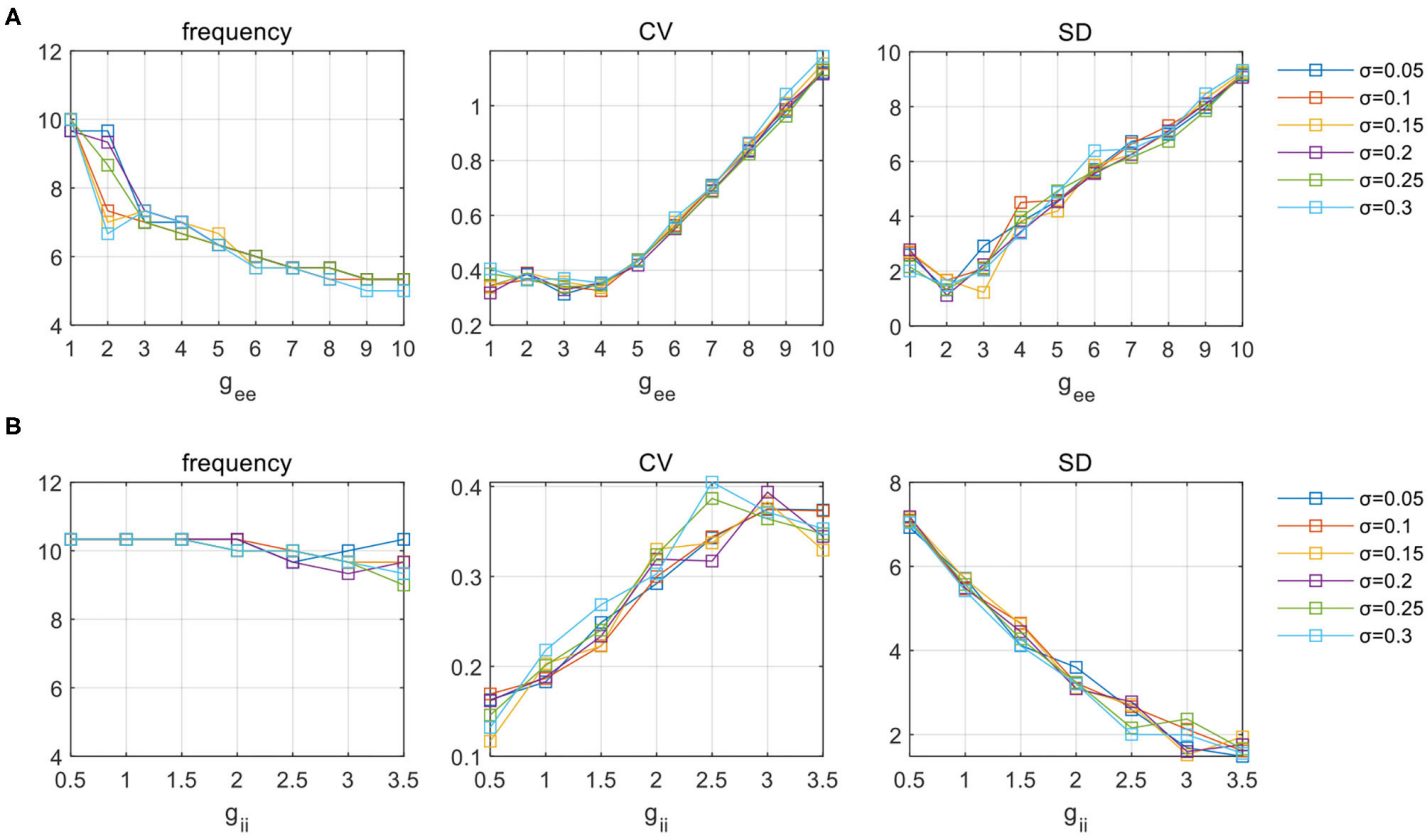
The influence of parameter **(A)** *g*_ee_ and **(B)** *g*_ii_ on network activities under six levels of deviation in parameter distribution. Network oscillatory behaviors are indicated in terms of frequency, CV and SD, respectively.

Additional tests are performed in the same way as having been stated in Sections 3.1 and 3.2. In Figure 9, we present the influence of heterogeneity under a specific parameter set for clarification (*g*_AMPA_ = 0.5, *g*_ie_ = 0.5). Figure 9A shows the variation of *g*_ee_ on network activities (measured by frequency, CV, and SD, respectively) under various levels of deviations. Six levels of heterogeneity with σ ranging from 0.05 to 0.3 at a step of 0.05 are superimposed on each subplot. It is obvious that the distributed couplings have limited effect on these metrics, and do not affect the evolution trend of network dynamics. Similar observations can be obtained under a varying *g*_ii_ given by Figure 9B. Tests performed under other parameter sets of E-I couplings are not shown but the findings are all the same. Therefore, it is safe to conclude that the simulation results obtained from the above sections display a considerable level of robustness which is amenable to parameter heterogeneity.

## 4. Conclusion and Discussion

In this study, we used a hybrid E-I network as a conceptual model of the hippocampus. We mainly focused on the dynamics of synchronous theta oscillations. Specifically, biological constraints regarding cell-type specific properties and network connectivity were considered to enhance the model's biological relevance. Both synchronous spike and burst patterns were observed in our simulations. Through careful parameter exploration, the distinct role of synaptic parameters in theta rhythm generation, modulation of synchronization and induction of burst patterns were identified. The main results are summarized as follows.

First, theta oscillations are found to be supported by either weak E(AMPA)-I couplings or strong NMDA current in our model. We further confirmed that the observed theta rhythm is mainly supported by the presence of slow NMDA current. In both regimes, counter-inhibition has little effect on regulating network activities.

Second, the decline of counter-inhibition can facilitate network synchronization in both regimes as indicated by an increasing level of overall SD. Specifically, in the weak AMPA regime, rhythmic oscillations are favored by a low level of counter-inhibition together with strong inhibition from I to E groups. Networks with SD <2.5 are thought to have no rhythmic activities, which occur in the regimes of weak I-E and strong E-I couplings. While in the strong NMDA regime, network synchronization is dominated by NMDA mediated excitatory connections.

Finally, it is revealed that recurrent excitation among PYs is remarkably effective in inducing burst synchronization in both regimes. We performed the exploration under a pathological setting to mimic pyramidal sprouting. The degree of burst activities was quantified by the average coefficient of variation of all PYs. SD was computed as well to confirm the enhancement of network synchronization. In the weak AMPA regime, the effect of *g*_ii_ on modulating network dynamics is mitigated by the dominant role of *g*_ee_ in E/I balance. The above results were all reconfirmed when parameter heterogeneity is introduced.

Computational models are increasingly employed to help understand the mechanisms of brain activities (Sejnowski et al., 1988). As brain activities span across multiple spatial and temporal scales, computational models can also be at various levels of complexity (Deco et al., 2008). Models of brain rhythms can either be as biophysically realistic as a detailed representation of physical structure (Bezaire et al., 2016), or conceptually represented by coupled neuronal oscillators that aim at revealing the essential mechanisms underlying observed activities (Kopell, 2005). Most previous modeling studies of theta rhythms use biophysically realistic models and focus on contributions of cellular mechanisms based on the Hodgkin-Huxley type of neurons (Rotstein et al., 2005; Kiss et al., 2006). The coherence of interneurons is related to theta frequency firing. A network consists of multi-compartment pyramidal, basket, and oriens-lacunosum moleculare (OLM) cells can also generate theta oscillations (Neymotin et al., 2011). However, since all the computational models are low-dimensional representation of high-dimensional reality, the necessity for them to be of that level of complexity is doubted, and insights found from less-detailed models may be more inefficient in unveiling the key mechanisms underlying biological systems (Kopell, 2005).

Mesoscopic phenomenological network with neuronal and synaptic interactions explicitly expressed is at a compromise of modeling complexity and cost of computation resources, while still enlightening in revealing the basic rules governing biological systems. Therefore, we chose to construct the CA1 network using the Izhikevich model as its dynamics can be experimentally constrained and specified to realistic neurons (Izhikevich, 2007), thus being a good conceptual representation of the hippocampal structure. Though random topology is a simplified form for network connectivity, the relative connection densities between neural populations are decided based on neurophysiological evidence found in hippocampal CA1. Specifically, PY neurons of hybrid dynamics are considered, and preferential connections are represented between PY and PV+ populations as revealed by experimental findings (Lee et al., 2014). All these considerations aim to enhance the biological relevance of the model. Nevertheless, our network is not designed to reproduce detailed biological data, so that network results are not interpreted specific to structural entities, but in a more general way, such as the relationship between E-I balance and network dynamic patterns.

The proposed network has acted as a structural substrate based on which network dynamics are explored. Physically, rhythms can originate from synchronous phenomena that can be represented by the synchronization of oscillators in physical models. To link network synchronization with physiological and pathological rhythmic oscillations in the hippocampus, parameter exploration was performed in two different ways, and this led to the observation of network dynamics of synchronous spike and burst patterns. Similar to experimental settings, network output is evaluated to inform network dynamics. Complex network models that study synchronization theoretically use the complex order parameter to quantify the level of synchronization over all neurons (Arenas et al., 2008). But in a hybrid network of two populations of PY neurons, measures of phase synchronization cannot produce a good indication of oscillatory behavior. Instead, we only focus on the averaged activity and use its SD to imply the overall degree of synchronization. SD is effective in implying network synchronization as a larger fluctuation of LFP can only be achieved when more neurons fire coherently. A similar strategy is employed by previous studies that use the mean field potential as a global parameter to represent the level of synchronization (Qu et al., 2014). In the present study, a threshold is used to distinguish between synchronous oscillatory behavior and non-rhythmic activities. The choice of the threshold is mainly based on visual inspections of network firing patterns. Among the synchronous oscillatory scenarios determined by SD, in the second part of the results, we further use CV to pick out the burst firing patterns. The threshold of CV is chosen as suggested in Börgers (2017b). We have to highlight that since network firing patterns vary quite smoothly under different SDs or CVs, the parameter boundaries as a result of thresholds are somewhat arbitrary. Therefore, the interpretation of results is made not to focus on the exact boundaries but the generic trend implicated by the pattern.

Synaptic factors related to network E-I balance are the main focus of this study. A previous study of hippocampal theta rhythm has used experimentally constrained Izhikevich neurons to clarify parameter regions that support theta rhythm generation (Ferguson et al., 2017). The exploration on E-I couplings shows similar scenarios for the existence of synchronous theta oscillations as in our results, where theta rhythms are largely lost when *g*_AMPA_ is too small, and network frequency increases as the reciprocal couplings between E-I groups become stronger. This consistency in findings has revealed some general rules of E-I networks for rhythm generation. As is shown in both results, sufficient inhibition from I to E is necessary for the network to synchronize. The indispensable role of fast inhibition in theta rhythms is experimentally and computationally verified (Wulff et al., 2009). In addition, simulations also demonstrate the significance of NMDA current in regulating network frequency. It has been reported that ablation of NMDA receptors on PV+ interneurons could lead to diminished theta oscillations in the hippocampus (Korotkova et al., 2010). In addition to the effect of E-I couplings, our results have emphasized the crucial role of counter-inhibition (*g*_ii_) in favoring network synchronization. Hippocampal PV+ cells are known to make potent connections to other inhibitory interneurons, including themselves. This special type of connection is a relatively rare connectivity in neural systems (Paz and Huguenard, 2015). Intuitively, a decline of *g*_ii_ will shift the network E/I balance to a more inhibiting side. Once the inhibitory firings resume, the effect of feedback inhibition becomes prominent so that rhythmic firings among PYs are probably shaped to be more regular. Meanwhile, burst synchronization can be induced in experimental models of seizure-like activities, which are mainly associated with recurrent excitatory interactions between hippocampal pyramidal cells. Similar network dynamics is initiated by increasing the corresponding parameter of *g*_ee_, which has demonstrated the explanatory power of our model network. We noticed that the transition into burst dynamics can be induced under a smaller *g*_ee_ as *g*_ii_ increases. Since an increase of *g*_ii_ equals an elevation in network excitation, we may attribute the occurrence of burst synchronization to hyperexcitation in terms of E/I balance. Besides synaptic gains, other factors such as synaptic time constants, sub-threshold oscillations, or post-inhibitory rebound spiking in pyramidal neurons may also be important for hippocampal theta oscillations (Stark et al., 2013), which remain to be studied in future work.

The proposed network is just a simplified representation for hippocampal oscillatory activities, in terms of both neuron models and network connectivity. This is because analysis on a biologically detailed whole-brain model to date is still far from realization, while several global projects have indeed been devoted to bridging the gap (Markram, 2012). In addition, our network also differs from a pure theoretical framework in a way that some biological facts are incorporated to interpret the results in a more physiological-related context. Further improvements can be achieved by containing more biological mechanisms of interest, such as gap junction or synaptic plasticity that are reported to be important in shaping network dynamics (Gigout et al., 2006; Igarashi, 2015). From a theoretical perspective, a detailed relationship between E/I balance and network dynamics should be further examined. We hope that our network can serve as a useful template for more hippocampal-related neural mechanisms to be studied.

## Data Availability Statement

The original contributions presented in the study are included in the article, further inquiries can be directed to the corresponding author.

## Author Contributions

ZL, QW, and FH conceived and performed the research as well as wrote the paper.

## Funding

This research was supported by the National Natural Science Foundation of China (Grants Nos. 11932003 and 11972115).

## Conflict of Interest

The authors declare that the research was conducted in the absence of any commercial or financial relationships that could be construed as a potential conflict of interest.

## Publisher's Note

All claims expressed in this article are solely those of the authors and do not necessarily represent those of their affiliated organizations, or those of the publisher, the editors and the reviewers. Any product that may be evaluated in this article, or claim that may be made by its manufacturer, is not guaranteed or endorsed by the publisher.
